# The Bleeding Must Be Stopped! Management in the Situation of Hemorrhages—A Prospective Randomized Controlled Prospective Simulation Study

**DOI:** 10.3390/healthcare12232370

**Published:** 2024-11-26

**Authors:** Michal Starosolski, Alicja Kalemba, Cezary Kaplan, Karolina Majewska, Krzysztof Ulbrich

**Affiliations:** 1 Emergency Medicine Department, Medical University of Silesia, 40-055 Katowice, Poland; krzysztof.ulbrich@sum.edu.pl; 2 Student Scientific Society at the Department of Emergency Medicine, Medical University of Silesia, 40-055 Katowice, Poland; alicja.kalemba@wp.pl (A.K.); cezary.kaplan@gmail.com (C.K.); 3 Department of Gastrointestinal Surgery, Medical University of Silesia, 40-055 Katowice, Poland; karolina.majewska@sum.edu.pl

**Keywords:** trauma, bleeding, tourniquet, bleeding wounds and injuries, blood loss, hemorrhage

## Abstract

Introduction: In a pre-hospital setting, uncontrolled hemorrhages in patients are undoubtedly a highly stressful situation for the rescuing medic, demanding prompt intervention. The effective stopping of a hemorrhage significantly reduces the risk of death for victims. The purpose of this study is to evaluate the students’ chosen methods and the effectiveness of their actions during a simulated hemorrhage situation. In addition, the students’ behavior and their cognitive overload classified using the NASA scale were also analyzed. Methods: The study enrolled 101 medical students, who were randomized and assigned sequentially to a study group (*n* = 49) and a control group (*n* = 52). The research group participated in a training course. Both groups then proceeded to a simulated hemorrhage scenario in a patient in a pre-hospital setting. Finally, the students assessed their cognitive overload using the NASA scale. Results: After the training, more students have achieved effective bleeding control (36/49, 73.5% vs. 22/52, 42.3%, *p* = 0.002). The prevalent method of bleeding control in both groups was using a tourniquet (97/101, 96%) with or without additional gauze and bandages. A significant improvement in the technique of the tourniquet application was observed—in the control group, 23/52 students (47.9%) applied it properly, while in the study group, it was 36/49 (73.5%). This resulted in a shorter time to achieve effective bleeding control and lower blood loss in the study group (*p* = 0.013 and *p* = 0.034, respectively). The median blood loss was 32 mL (IQR = 24, range 11–65 mL) in the study group compared to 53 mL (IQR = 26, range 10–117 mL) in the control group. Conclusions: The education program for young medics needs changes, including an increased emphasis on learning procedures and improving practical skills.

## 1. Introduction

Europe’s political and geographic situation has changed significantly in recent times. This has highlighted new challenges, especially for medical professionals in pre-hospital care [1]. Unintentional injuries are one of the most common causes of death. In the U.S., such injuries are the third cause of death [2]. Trauma patients are exposed to several complications that worsen their prognosis, both in the short-term and long-term aspects. The three most important aspects—hypothermia, acidosis, and coagulopathy—are known as the triad of death [3]. It confirms that the 24 h mortality of trauma patients increased with the addition of all three death triad components [4]. It should be stated that massive external hemorrhages affect all components of the triad of death. Guidelines from scientific societies, i.e., Advanced Trauma Life Support (ATLS) [5], Pre-Hospital Trauma Life Support (PHTLS) [6], and International Trauma Life Support (ITLS) [7], indicate that the most important management (immediately following the rescuer’s safety assessment) at the scene should be to stop visible massive external bleeding as soon as possible. Nowadays, many hemostatic agents can be used to control bleeding. One of them is a tourniquet that can be placed on both the upper and lower limbs. A limb tourniquet can safely control bleeding without an increased risk of significant complications [8]. Moreover, using a tourniquet seems more effective and faster than building a pressure dressing. Massive bleeding within the pelvic and shoulder girdle should be treated with the “wound packing” technique using dedicated hemostatic dressings. Dedicated tourniquets designed to control bleeding within the shoulder and pelvic girdle or abdominal aortic tourniquets also raise hope; however, at the time of writing the article, they were not available for use in civilian medicine [9]. Besides bleeding control, pre-hospital management with trauma patients should include a full physical examination in the ABCDE algorithm, rapid trauma surveying, and performing critical life-saving interventions, such as opening the airway, using a pelvic binder, or decompressing a tension pneumothorax. Moreover, it is necessary to provide the patient with adequate and effective analgesia, even with the use of opioid drugs or ketamine [10], and to take care of the patient’s thermal and mental comfort, including dedicated thermal blankets and the transfusion of warm fluids. It should be mentioned that one layer of thermal blankets is highly insufficient to prevent posttraumatic hypothermia. As far as fluid therapy is concerned, massive fluid transfusion may lead to a “pop the clot” phenomenon, which results in increasing bleeding. That is why fluid therapy should be conducted with the principles of permissive hypotension [11] or until the presence of a peripheral pulse. Furthermore, fluid resuscitation should be provided with crystalloid fluids [12]; some research undermines this statement [13]. However, hemorrhages resulting from trauma remain the leading cause of preventable deaths [14]. The Hartford Consensus and the Stop The Bleed campaign, which targeted the civilian sector, certainly contributed to reducing preventable deaths [15]. The American College of Surgeons “Bleeding Control Basic” (B-Con) course was implemented as part of the campaign. Its goal is to make ordinary emergency responders competent in applying tourniquets to stop bleeding in extremities. To this end, according to available data, correctly applied tourniquets had an increased survival rate [16]. In addition, the Hartford Consensus states that when a commercial tourniquet is unavailable, an improvised tourniquet can be made and used, while the B-Con course does not address this issue [17].

The stressful situations that require rapid response greatly increase medical professionals’ cognitive overload, especially those without significant experience. An objective assessment of perceived overload would be impossible without a standardized rating system, namely the NASA Task Load Index. This tool was initially developed mainly in flight trainings. However, due to its functionality, it has been successfully implemented into medicine to assess procedural workload [18]. The scale is used to evaluate perceived task load immediately after the performed task through mental, emotional, and physical dimensions.

Our study aimed to evaluate the behaviors and effectiveness of actions displayed by medical students during a hemorrhage simulation. The cognitive overload of the participating students was rated using the NASA scale.

## 2. Materials and Methods

The study was prospective in design and conducted in the Education and Medical Simulation Center, Medical University of Silesia, Katowice, on September 5th and 9th, 2022. It included 5th- and 6th-year medical students (Medical Doctor, MD) of the Faculty of Medical Sciences in Katowice. All participants voluntarily gave written consent to participate in the study. The students did not derive any advantages from participating in the study. The study was not subject to evaluation by a bioethics committee because it was performed on simulators.

The SimMan 3G (Laerdal Medical AS, Stavanger, Norway) was used in the study, including the left leg amputee overlay. Participants were also provided with a tourniquet (CAT-Combat Application Tourniquet NSN 6910-01-560-2972 -Trainer Tourniquet, Rock Hill, SC, USA), a restraint, gauze pads, elastic bandages, and knitted bandages (in any quantity).

The students were assigned to a control or research group before participating in the simulation. The study used simple randomization using The Research Randomizer (a freeware tool available at www.randomizer.org). The research group attended a lecture designed to provide basic information on securing a trauma patient in a pre-hospital setting. Participants were also shown wound-packing techniques and the effective application of a CAT tourniquet. The information presented was based on ITLS and the B-Con course. During the conducted lecture, the lecturer did not suggest to the participants what awaited them in the next part of the study. Subsequently, a simulation based on a 3 min scenario was conducted in both groups. The scenario allowed the participants to impersonate an Emergency Medical Service member tasked with securing a trauma patient in a pre-hospital setting. The scenario was based on the element of surprise—a hemorrhage, which was meant to stress the participant. The simulation began the moment the participant entered the room where the patient was located. The goal of the simulation was to recognize the hemorrhage from an amputated left lower leg in an already confused patient and to stop it successfully. To perform this task, the student had the following at their disposal, prepared by another member of the Emergency Medical Service in a place visible to the participant: a tourniquet, restraint, gauze pads, elastic bandages, and knitted bandages. The simulation ended when the bleeding was successfully stopped or when the scenario time expired. The design of the scenario allowed the participant to focus only on stopping the bleeding. The participant was assured that the patient did not require him to do other activities.

After the simulation, all participants evaluated their cognitive overload using the NASA scale. This is a publicly available, validated psychometric tool for assessing the subjective level of perceived workload. The tool consists of six 20-point visual analog scales with opposing descriptors at the ends (high/low). The tool generates an overall measure of workload resulting from an assessment of mental (i.e., remembering, considering, deciding), physical, and time demands, as well as the operator’s frustration, effort, and productivity during that time [19].

The statistical analyses were performed using IBM SPSS Statistics 29. Quantitative variables were expressed as means with standard deviations (SD) or medians with interquartile ranges (IQRs). Qualitative variables were given as absolute values and percentages. A *p* value of <0.05 was considered statistically significant. The Shapiro–Wilk test of normality was applied to verify the distribution of the quantitative variables. The homogeneity of variance was tested using Levene’s test. Between-group differences were verified using the chi-square test or Fisher’s exact test for qualitative variables and Student *t*-test or the Mann–Whitney test for quantitative variables.

## 3. Results

In total, 101 students (28%, 28 men) were included; 49% (49/101) were in the study group and 51% (52/101) were in the control group. Most of the students were at the beginning of their fifth year of medical studies (56/101, 55%).

**Figure 1:**
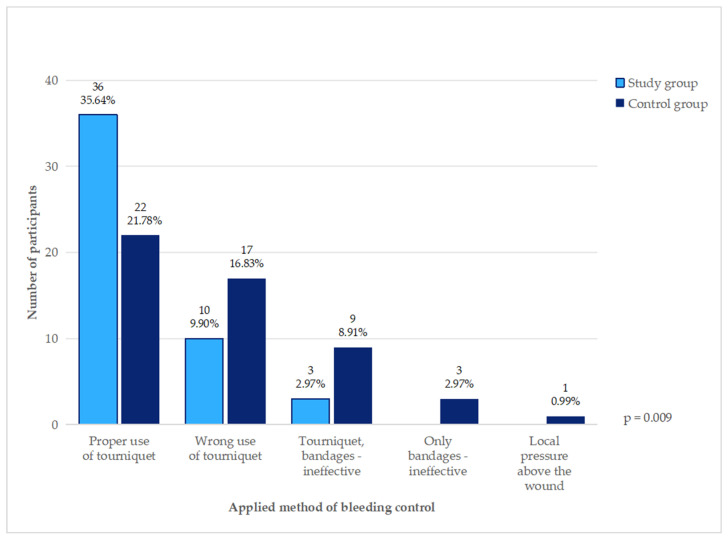
Hemorrhage control methods applied by students.

After the training, more students achieved effective bleeding control (36/49, 73.5% vs. 22/52, 42.3%, *p* = 0.002). The detailed results are presented in Table 1. The prevalent method of bleeding control in both groups was using a tourniquet (97/101, 96%) with or without additional gauze and bandages. Methods of bleeding control used in both groups are presented in Figure 1. A significant improvement in the technique of the tourniquet application was observed—in the control group, 23/52 students (47.9%) applied it properly, while in the study group, it was 36/49 (73.5%). After the training, there were fewer crucial mistakes, like applying the tourniquet distal to the wound or not twisting the rod. This resulted in a shorter time to achieve effective bleeding control and lower blood loss in the study group (*p* = 0.013 and *p* = 0.034, respectively). The median blood loss was 32 mL (IQR = 24, range 11–65 mL) in the study group compared to 53 mL (IQR = 26, range 10–117 mL) in the control group. Moreover, men were better than women in terms of the effective use of tourniquets, regardless of the training (23/27, 85.2% vs. 36/70, 51.4%, *p* = 0.005, no interaction between training and gender observed).

Regarding the NASA scale, a significant difference was observed only in the performance score, which was higher in the control group (median 40 vs. 20, *p* = 0.036).

## 4. Discussion

The lecture conducted among the participants in the research group allowed them to take more effective actions during the hemorrhage simulation compared to the control group. Students in the control group rated their performance significantly better than students in the research group.

In accordance with our assumptions, the students in the research group performed better on the task in general. From one perspective, this is a desirable effect. In contrast to the study, our work shows that the implementation of training brings a real increase in efficiency [20]. In addition, it directly stimulates the learning curve [16,21]. On the other hand, this is unfortunately a very worrying phenomenon. Students who will become full-fledged medics in the near future have a problem with stopping hemorrhage. We cannot exclude that the forced break in classes caused by the SARS-CoV-2 epidemic is indirectly responsible for such a result. In addition, the reduced ratio of practical classes in the study plan may also be responsible for this. The study shows that knowledge and practice are inconsistent and insufficient, even among surgical trainees [22]. Perhaps OSCE training introduced in England, among other countries, can help reduce practice deficiencies. In the survey, students rated OSCE training as necessary in terms of practical skills. They mentioned that after the test in this form, they felt more confident and prepared for their work [23]. Another study shows that OSCE training conducted by upper-year students for younger ones results in mutual benefits. Both junior and senior students were able to identify and reduce their knowledge deficits in this way [24]. The authors make a far-reaching conclusion that may suggest deficient bleeding control in other medical professions, including nurses and midwives. However, more research among these medical majors, especially in the European literature, is lacking to formulate this conclusion fully. Despite the training provided to the study group, there were some inadequacies in stopping bleeding. This is unequivocal about the continued need for more training. This is also evident from this study [25]. This, unfortunately, comes at a high cost. However, the study shows that even a mobile app can positively influence the skills of putting on a tourniquet [26]. It should be noted that in this study, those using the mobile app were after the B-Con course. Consolidating the experience gained from the workshop and synthesizing the knowledge included in the mobile app is an excellent way to teach. This will optimize costs and a longer retention of knowledge in medics. Another disturbing phenomenon our work highlighted in the control group is the Dunning–Kruger effect [27]. This is a cognitive bias because unskilled people overestimate their skills in that area, while highly skilled people underestimate their skills. The study shows that this effect mainly affects first-year students [28]. In addition, female students, the general population of students without academic achievement, with fewer hours of teaching, or more sleep, are more predisposed to develop this effect. The Dunning–Kruger effect may contribute to the deterioration of patient services in the future. Therefore, as mentioned earlier, we agree with the article’s authors about taking quality-enhancing educational measures to minimize the Dunning–Kruger effect.

In our study, we used the NASA scale to determine the cognitive workload during a hemorrhage simulation scenario to measure effort, frustration, performance, mental demand, physical demand, and temporal demand in student subjects. The definite advantage of this tool is its standardization and very low inter-individual variability due to a category weighting system that takes into account the strengths and weaknesses reported by the individual [18]. An additional advantage is the public availability of this tool. Unfortunately, the scale also has disadvantages. One of them is the length and time required to complete this survey. The scale questionnaire is composed of phases, and it took more time to complete than the scenario conducted. We suppose that the length of completion may have reduced participants’ concentration and indirectly influenced falsified self-assessments. Cognitively overloaded students for whom the tool was a complete novelty may have chosen thoughtless answers out of a desire to complete the survey quickly.

Our study showed that stopping bleeding was less effective among female participants. This could certainly be due to their physical conditions. However, this phenomenon should be further studied.

The study concluded that current devices need to be more complex for everyone to use them correctly [29].

The study found that the quality of the tourniquets also affects the final success when trying to stop bleeding [17]. CAT tourniquets compared to other available tourniquets were characterized by higher pressure, reduced blood loss, and speed of application. As a result, there is a need to standardize the tourniquet design to reduce incidents where the tourniquet, by its design, will be the weakest element.

## 5. Conclusions

In the face of new risks, changes in the teaching plan of medical students from civil universities are needed. This will expand the range of practical skills among future doctors and reduce the scale of cognitive error. Classes conducted using the medical simulation method provide the opportunity for better preparation for the profession in a shorter time than traditional education. Simulations provide students with very good conditions for practicing and checking the level of acquired clinical skills, both technical and non-technical, while at the same time avoiding risk to the patient [30,31].

## 6. Limitations

Our work is not without limitations. The main limitation of our work is the small size of the study population. The organizational difficulties and the fact that only volunteer students participated in the study precluded the possibility of assembling a population that was quantitatively as representative as possible. One of them is the limitations of the simulation and its scenario. Certainly, the simulation can induce a certain stress level in the participants. However, it is not the same stress experienced by doctors who work with such difficult cases daily. The study we conducted mainly focused on performing one activity, which was securing an actively bleeding limb. For this reason, the participants could not experience the burden of responsibility and the consequences of their own decisions when saving a human life. We are convinced this significantly affected the answers given in the NASA form. Another limitation of our study was the numerical disproportion in terms of the participants’ gender. However, we believe that this only affects our gender-oriented conclusions. Due to the generally increased ratio of women to men in medical studies and the random selection of participants, we would not have been able to prevent the disproportion. Another area for improvement is the possible difference in the level of knowledge and experience of the participants. This could directly impact their effectiveness during the simulation.

## Figures and Tables

**Table 1:** Comparison of the study group and control group.

	Study Group (*n* = 49)	Control Group (*n* = 52)	*p* Value
Year of study			0.233
5th	24 (49%)	32 (61.5%)	
6th	25 (51%)	20 (38.5%)	
Female sex	36 (73.5%)	37 (71.2%)	0.827
Effective bleeding control = proper use of tourniquet	36 (73.5%)	22 (42.3%)	0.002
Methods of bleeding control			0.009
Tourniquet (effective)	36 (73.5%)	22 (42.3%)	
Tourniquet—wrong technique	10 (20.4%)	17 (32.7%)	
Tourniquet + bandages/gauze—ineffective	3 (6.1%)	9 (17.3%)	
Only bandages/gauze—ineffective	0	3 (5.8%)	
Local pressure above the wound	0	1 (1.9%)	
Use of tourniquet	49 (100%)	48 (92.3%)	0.118
Effective	36/49 (73.5%)	23/48 (47.9%)	0.013
Windlass rod twisted insufficiently	5/49 (10.2%)	12/48 (25%)	
Windlass rod not twisted	1/49 (2%)	2/48 (4.2%)	
Applied below the wound	0	1/48 (2.1%)	
Blood loss (mL)	32 (24)	53 (26)	0.013
Time to effective bleeding control (minutes)	1.07 (2.17)	3.00 (2.15)	0.034
NASA scale			
Mental demand	55 (43)	53 (40)	0.827
Physical demand	55 (40)	53 (35)	0.705
Temporal demand	65 (45)	70 (25)	0.283
Performance	20 (45)	40 (34)	0.036
Frustration	50 (48)	48 (55)	0.731
Effort *	55.1 (18.81)	52.3 (17.39)	0.44

* Mean and standard deviation are presented. For all other quantitative variables, the median and interquartile range are presented.

## Data Availability

The original contributions presented in the study are included in the article, further inquiries can be directed to the corresponding author.
